# Preoperative Nutritional Status as a Risk Factor for Major Postoperative Complications Following Anterior Lumbar Interbody Fusion

**DOI:** 10.1177/2192568218760540

**Published:** 2018-03-18

**Authors:** Chierika O. Ukogu, Samantha Jacobs, Willliam A. Ranson, Sulaiman Somani, Luilly Vargas, Nathan J. Lee, John Di Capua, Jun S. Kim, Khushdeep S. Vig, Samuel K. Cho

**Affiliations:** 1 Icahn School of Medicine at Mount Sinai, New York, NY, USA

**Keywords:** NSQIP, ALIF, lumbar interbody fusion, outcomes, complications: multi-institutional, multivariate logistic regression model

## Abstract

**Study Design:**

Retrospective study.

**Objectives:**

To determine rates of medical and surgical postoperative complications in adults with hypoalbuminemia undergoing anterior lumbar interbody fusion (ALIF).

**Methods:**

This was a retrospective analysis of prospectively collected data from the American College of Surgeons National Surgical Quality Improvement Program (ACS-NSQIP) database of patients (≥18 years old) undergoing ALIF procedures, identified by CPT (Current Procedural Terminology) code from 2011 to 2014. Poor nutritional status was defined by a preoperative serum albumin level <3.5 g/dL, and albumin levels above this were considered normal. Multivariate logistic regression models were utilized to assess preoperative risk factors including nutritional status as predictors of specific postoperative complications. Significance was defined as *P* < .05 and odds ratios (ORs) were calculated with a 95% confidence interval (CI). This model was used to determine the strength of nutritional status as an adjusted predictor of adverse postoperative events.

**Results:**

There were 3184 ALIF cases, including 1,275 (40%) of which had preoperative serum albumin levels. 53 (4.15%) patients were classified as having poor nutrition status. Poor preoperative nutritional status was shown to be a strong independent predictor of length of stay ≥5 days (OR = 2.56, 95% CI 1.43-4.59, *P* = .002), urinary tract infection (OR = 5.93, 95% CI 2.11-16.68, *P* = .001), and sepsis (OR = 5.35, 95% CI 1.13-25.42, *P* = .035) compared to patients with normal preoperative serum albumin levels.

**Conclusions:**

Our analysis shows that patients with poor nutritional status before ALIF are independently at risk for sepsis as well as increased length of stay and urinary tract infection.

## Introduction

Anterior lumbar interbody fusion (ALIF) is used to treat various lumbar degenerative pathologies, including degenerative disc disease, recurrent disc herniation, and sagittal imbalance.^1^ Current data suggests that the complication rate following ALIF ranges anywhere from 4% to 26%.^1,2^ Given such a wide range of potential complications following ALIF procedures, it is important to identify risk factors that can be modified prior to surgery in an effort to mitigate postoperative risks.

Preoperative serum albumin levels have been an established marker for overall nutrition, and hypoalbuminemia, measured by an albumin level less than 3.5 g/dL can be used as a proxy for malnourishment.^3^ A growing corpus of evidence within orthopedics has linked malnutrition to increased risk for complications following surgery.^4–7^ These risk factors have also been described in other spinal surgeries such as posterior cervical fusion.^2,8^

Until now, such studies have not been conducted for patients undergoing ALIF procedures, even though evidence suggests malnutrition appears to impact spinal fusion surgery.^8–10^ Therefore, the present study uses the American College of Surgeons National Surgical Quality Improvement Program (ACS-NSQIP) database to illuminate possible links between preoperative nutrition status and 30-day postoperative complications in ALIF procedures.

## Materials and Methods

This study received an exemption by the institutional review board of the Icahn School of Medicine at Mount Sinai.

### Data Source

This was a retrospective study of prospectively collected data in the 2010-2014 ACS-NSQIP database. ACS-NSQIP is a large national database with risk adjusted 30-day postoperative morbidity and mortality outcomes. More than 500 hospitals that vary in size, socioeconomic location, and academic affiliation contributed data to the 2010-2014 ACS-NSQIP database.^11^ ACS-NSQIP data is collected prospectively by dedicated clinical abstractors at each institution on more than 150 demographic, preoperative, intraoperative, and 30-day postoperative variables.^11^

### Patient Selection

This was a retrospective analysis of prospectively collected data from the ACS-NSQIP database of patients undergoing ALIF procedures between 2011 and 2014. Surgical cases were included for patients aged ≥18 years with Current Procedural Terminology (CPT) coded for anterior or lateral lumbar interbody fusion (22 558). Adults undergoing nonelective surgery, emergency surgery, and those with current pneumonia, current sepsis, current pregnancy, wound class >1, or a previous operation within 30 days of the principal operation were excluded from the study. Patients were then subdivided based on nutritional status. Those with poor nutritional status were among those with preoperative serum albumin levels of <3.5 g/dL. All other patients were considered normal, and those without preoperative serum albumin levels were excluded from the analysis.

### Variable Definitions

Preoperative variables included race (Caucasian, African American, Hispanic, and “Other”). “Other” race category included American Indian, Alaska Native, Asian, Native Hawaiian, Pacific Islander, or Unknown/Not Reported. Other demographic variables included were gender, obesity (≥30 kg/m^2^), diabetes (non–insulin-dependent diabetes mellitus or insulin-dependent diabetes mellitus), current smoking (within 1 year of surgery), dyspnea (≤30 days prior to surgery), and functional status prior to surgery (independent or partially/totally dependent ≤30 days prior to surgery). Preoperative comorbidity variables were also used in this analysis: pulmonary comorbidity (ventilator dependent ≤48 hours prior to surgery or history of chronic obstructive pulmonary disease ≤30 days prior to surgery), cardiac comorbidity (use of antihypertensive medication or history of chronic heart failure ≤30 days prior to surgery), renal comorbidity (acute renal failure ≤24 hours prior to surgery or dialysis treatment ≤2 weeks prior to surgery), steroid use for chronic condition (≤30 days prior to surgery), ≥10% loss of body weight (in the past 6 months), bleeding disorder (chronic, active condition), preoperative transfusion of ≥1 unit of whole/packed red blood cells (RBCs) (≤72 hours prior to surgery), ASA (American Society of Anesthesiologists) class ≥3. Thirty-day postoperative outcome variables include mortality, wound complication (deep surgical site infection, organ space infection, or wound dehiscence), pulmonary complication (pneumonia, unplanned reintubation, or duration of ventilator-assisted respiration ≥48 hours), venous thromboembolism (pulmonary embolism or deep vein thrombosis), renal complication (progressive renal insufficiency or acute renal failure), urinary tract infection (UTI), cardiac complication (cardiac arrest requiring cardiopulmonary resuscitation myocardial infarction), intra-/postoperative RBC transfusion, reoperation (related to initial procedure), prolonged length of stay (≥10 days), and unplanned readmission (related to initial procedure). The ACS-NSQIP guide may be consulted for further explanations of variables.

### Statistical Analysis

Descriptive and comparative statistics of demographics, comorbidities, operative details, and postoperative complications were analyzed for all patients. In the univariate analysis, categorical variables were assessed using Pearson chi-square or Fisher exact test where appropriate. Variables with *P* < .2 in the univariate analysis or those with established clinical relevance were carried forward into the multivariate analysis. This specific selection criterion was used to consider as many potential risk factors as possible without compromising the validity of regression models. Univariate and multivariate analysis was used to determine whether nutritional status was a predictor of postoperative complications after ALIF. Multivariate logistic regression analysis was also used to determine independent risk factors for 30-day complication rates after ALIF surgery. A *P* value <.05 was considered statistically significant. The overall model was assessed using the C-statistic, which is the area under the receiver operating characteristic curve. SAS software (Version 9.3, SAS Institute Inc, Cary, NC) was used for all statistical analyses. This model was used to determine the strength of nutritional status as an adjusted predictor of adverse postoperative events. All multivariate analyses controlled for by demographic and comorbidity variables are included in Table 1.

**Table 1.** Univariate Analysis of Patient Demographic, Preoperative, and Intraoperative Variables Between Nutritional Status (N = 1275).

Category	Albumin ≥3.5 g/dL		Albumin <3.5 g/dL		*P*
	n	%	n	%	
Sex					
Female	670	54.8	33	62.3	.287
Male	552	45.2	20	37.7	
Race/Ethnicity					
White	1059	86.7	44	84.6	.090
Other	51	4.2	0	0.0	
Black	95	7.8	8	15.4	
Hispanic	17	1.4	0	0.0	
Age	377	30.9	18	34.0	.632
Obese	569	46.7	22	41.5	.460
Diabetes	165	13.5	11	20.8	.134
Dyspnea	60	4.9	5	9.4	.143
Functional status					
Independent	1188	97.6	51	96.2	.521
Partially or totally dependent	29	2.4	2	3.8	
Pulmonary comorbidity	96	7.9	9	17.0	.018
Cardiac comorbidity	620	50.7	29	54.7	.570
Renal comorbidity	4	0.3	0	0.0	.677
Smoking	286	23.4	19	35.9	.038
Steroid use	32	2.6	2	3.8	.610
Recent weight loss	4	0.3	0	0.0	.677
Bleeding disorder	14	1.2	4	7.6	<.001

## Results

A total of 3184 patients undergoing elective ALIF cases during 2011 to 2014 were identified in the NSQIP database. A total of 1275 (40%) of those had preoperative serum albumin levels included, and 53 (4.15%) patients were classified as having preoperative hypoalbuminemia (serum albumin <3.5 g/dL).

Comparison of patient demographic revealed that patients who had low serum albumin had higher rates of smoking history (*P* = .003). These patients also demonstrated higher rates of comorbidities, including chronic or active bleeding disorder (*P* < .001) and pulmonary comorbidity (*P* = .018). Finally, hypoalbuminemic patients had an ASA classification ≥3 more often compared with their counterparts with normal serum albumin (*P* = .007).

### Outcomes

Univariate analysis of serious adverse postoperative complications revealed length of stay ≥5 days (*P* < .001), wound complications (*P* = .017), pulmonary complications (*P* = .019), urinary tract infections (*P* < .001), intra-/postoperative RBC transfusion (*P* = .007), sepsis (*P* = .029), and unplanned readmission (*P* = .039) were significantly associated with preoperative albumin levels (Table 2). These complications were brought forward to the multivariate logistic regression performed to assess whether nutritional status, as measured by hypoalbuminemia, was the sole driver of postoperative complications following ALIF.

**Table 2.** Univariate Analysis of 30-Day Postoperative Outcomes Between Nutritional Status (N = 1275).

Category	Hypoalbuminemia		No Hypoalbuminemia		*P*
	n	%	n	%	
Mortality	2	0.2	1	1.9	.113
Length of stay ≥5 days	329	26.9	28	52.8	<.001
Wound complication	28	2.3	4	7.6	.017
Pulmonary complication	18	1.5	3	5.7	.019
Venous thromboembolism	19	1.6	0	0.0	.360
Renal complication	3	0.3	0	0.0	.718
Urinary tract infection	21	1.7	5	9.4	<.001
Cardiac complication	5	0.4	0	0.0	.641
Intra-/postoperative red blood cell transfusion	115	9.4	11	20.8	.007
Sepsis	10	0.8	2	3.8	.030
Reoperation	44	3.6	4	7.6	.140
Unplanned readmission	60	4.9	6	11.3	.039

A total of 1269 patients were used in the multivariate analysis. Poor preoperative nutritional status, among those with serum albumin levels <3.5g/dL was shown to be a strong independent predictor of length of stay ≥5 days (odds ratio [OR] = 2.56, 95% CI 1.43-4.59, *P* = .002), urinary tract infection (OR = 5.93, 95% CI 2.11-16.68, *P* = .001), and sepsis (OR = 5.35, 95% CI 1.13, 25.42, *P* = .035) rates compared to patients with normal preoperative serum albumin levels when controlling for patient demographic, intraoperative, and perioperative characteristics (Table 3).

**Table 3.** Multivariate Analysis of Nutritional Status as a Risk Factor for 30-Day Postoperative Outcomes Following ALIF (N = 1269).

Outcome	Nutritional Status	Odds Ratio	Lower CL	Upper CL	*P*
Length of stay ≥5 days	Albumin <3.5 g/dL vs albumin ≥3.5 g/dL	2.56	1.43	4.59	.002
Urinary tract infection	Albumin <3.5 g/dL vs albumin ≥3.5 g/dL	5.93	2.11	16.68	.001
Sepsis	Albumin <3.5 g/dL vs albumin ≥3.5 g/dL	5.35	1.13	25.42	.035

Abbreviations: ALIF, anterior lumbar interbody fusion; CL, confidence limit.

## Discussion

Poor nutritional status, measured by preoperative serum albumin levels is an independent risk factor for major postoperative complications. In this cohort, poorly nourished patients were more than 5 times more likely to develop postoperative sepsis and UTI, and they were 2.5 times more likely to remain in the hospital for 5 or more days following ALIF procedures. To our knowledge, no study has performed a multicenter analysis of this scale focusing on adults undergoing elective ALIF procedures. The present data confirms our hypothesis that poor preoperative nutritional status is linked to adverse postoperative outcomes.

Our key finding that sepsis and UTI are independently associated with preoperative hypoalbuminemia is supported by previous research on outcomes following ALIF procedures. A review of the NSQIP database for 30-day postoperative complications supported our findings concluding that UTI and sepsis were the most commonly observed complications following ALIF.^12^ While no prior research has looked at nutritional status and postoperative complications following ALIF, a comparable study indirectly assessed preoperative nutritional status with the validated modified frailty index (mFI), which combined 16 NSQIP variables, including serum albumin levels as a proxy for preoperative fitness.^13^ They studied 3920 patients undergoing elective ALIF from 2010 to 2014, and despite including hypoalbuminemia in their analysis, length of stay ≥5 days, UTI, or sepsis was found to be significantly associated with mFI score.^10,13–15^

Preoperative nutritional status among patients undergoing other spine surgery procedures also garnered similar findings. Recently, Lee et al^9^ found that preoperative nutritional status was an independent risk factor for length of stay >5 days, pulmonary complications, intra-/postoperative blood transfusions, sepsis, and venous thromboembolism in patients undergoing posterior cervical fusion. Similarly, Fu et al^16^ showed that nutritional status was independently associated postoperative pulmonary and cardiac complications, reoperation, and longer length of stay in patients undergoing anterior cervical discectomy and fusion. Bohl et al^17^ also came to similar conclusions for a cohort of 4310 patients undergoing posterior lumbar fusion from the ACS-NSQIP database. In this analysis, malnutrition independently predicted wound dehiscence, UTI, infections, increased length of stay, and readmission.^17^ Both studies used the ACS-NSQIP database and our findings further corroborate a development in the literature linking preoperative nutritional status and postoperative outcomes specifically regarding prolonged length of stay and sepsis.

Possible mechanisms for malnutrition have also been previously explored. Natural reduction in caloric intake is a well-described explanation for malnutrition in elderly patients.^18–20^ It is theorized that patients without preoperative hypoalbuminemia are ill equipped to cope with the nutritional demands of surgery leading to increased inflammation and prolonged wound healin,^21^ both of which are implicated in the pathway to sepsis and UTI. Prolonged length of stay has significant clinical and economic implications and could be explained by the aforementioned complications following ALIF. This theory is supported by published studies showing an independent association between recent weight loss resulting in malnutrition and an increased risk for postoperative complications.^22^ Conversely, studies have shown that obese patients undergoing spine surgery have increased risk for postoperative complications, but our cohort was shown to be malnourished and obese.^23,24^ Overall, these findings suggest that malnutrition both in the presence of weight loss or obesity with poor nutritional intake has a complex interaction with surgical morbidity.

Potential limitations of this study include the fact that the ACS-NSQIP database only follows complication rates to 30 days postoperatively. Additionally, the ACS-NSQIP database does not capture other established markers of nutrition like vitamin D levels or total lymphocyte count which could further explain the underlying etiology of postoperative sepsis, UTI, and prolonged length of stay risks. Additionally, both Fu et al^16^ and Lee et al^9^ describe possible selection bias for among patients that are chosen to undergo preoperative serum albumin levels. They posit that because such preoperative labs may not be standardized, physicians may be suspicious of underlying infection in the sample of patients with labs. Additionally, serum albumin is a sensitive surrogate marker for internal protein stores and may reflect an unidentified subclinical disease.^8,25,26^ Therefore, hypoalbuminemia itself may not be the causal agent of postoperative sepsis, prolonged length of stay, or UTI. Furthermore, our analysis may have underestimated the incidence of malnutrition among the members of this cohort with subclinical illnesses.

This current study is also limited by design as a retrospective analysis of prospectively collected data lending to selection bias. The NSQIP database itself is limited to the participating 512 hospitals and results may not represent nationwide trends. Furthermore, the NSQIP database limits us in understanding pathways that could be causing the low serum albumin such as increased metabolic demands caused by cancer or malnutrition due to weakness and inability to feed appropriately. CPT code 22 558 used to isolate ALIF procedures includes both anterior and lateral approach, and the NSQIP does not provide operative notes to differentiate between the 2 procedures. Another valid limitation to the study is the inability to delineate between patients’ reasons for undergoing ALIF. Though this cohort was limited to elective procedures only, we cannot further assess if patients opted for surgery due to issues like degenerative scoliosis or disc degeneration.

In conclusion, this study confirms that hypoalbuminemia is an independent predictor of postoperative complications for adults undergoing elective ALIF. Such findings add to the growing corpus of evidence bolstering the need for appropriate preoperative screening of nutritional status in order to mitigate devastating postoperative risk factors. Given the gravity of these adverse events, surgeons should consider using hypoalbuminemia as a prognostic tool to detect possible malnutrition or presence of systemic disease prior to operation.
